# The Selvester QRS score as an estimative of myocardial injury in acute chagasic patients from the Brazilian Amazon

**DOI:** 10.1186/s12879-021-06083-x

**Published:** 2021-04-29

**Authors:** Katia do Nascimento Couceiro, Jessica Vanina Ortiz, Michael do Nascimento Correia, Mônica Regina Hosannah da Silva e Silva, Alba Regina Brandão, Paula Rita Leite da Silva, Susan Smith Doria, Reinaldo Bulgarelli Bestetti, Débora Raysa Teixeira de Sousa, Rubens Celso Andrade da Silva Junior, Maria das Graças Vale Barbosa Guerra, João Marcos Bemfica Barbosa Ferreira, Jorge Augusto de Oliveira Guerra

**Affiliations:** 1 Programa de Pós Graduação em Medicina Tropical, Escola de Ciências da Saúde, Universidade do Estado do Amazonas, Manaus, Brazil; 2 Fundação de Medicina Tropical Dr. Heitor Vieira Dourado, Manaus, Brazil; 3 Departamento de Ciências Fisiológicas, Universidade Federal do Amazonas, Manaus, Brazil; 4 Escola de Ciências da Saúde, Universidade do Estado do Amazonas, Manaus, Brazil; 5 Fundação de Hematologia e Hemoterapia do Amazonas, Manaus, Brazil; 6 Escola de Medicina, Universidade Federal do Amazonas, Manaus, Brazil; 7 Escola de Medicina, Universidade de Ribeirão Preto, São Paulo, Brazil

**Keywords:** Chagas disease, Selvester score, Myocardial injury, Brazilian Amazon

## Abstract

**Background:**

In the Brazilian Amazon, a new epidemiological profile of Chagas disease transmission, the oral route, has been detected and cited as being responsible for the increase in acute cases in Brazil. The clinical evaluation of acute Chagas disease (ACD) has been a challenge since it can progress to a chronic phase with cardiac alterations, and the follow-up by modern diagnostic methods is very difficult due to the socio-geographical characteristics of the Brazilian Amazon. Thus, alternatives should be sought to alleviate this problem. We conducted a study to evaluate subjects with ACD using the 12-lead ECG QRS score (Selvester score) as an estimative of myocardial injury progression before and after ACD treatment.

**Methods:**

The study included indigenous subjects from the Amazon region with ACD in clinical follow-up at the Fundação de Medicina Tropical Dr. Heitor Vieira Dourado (FMT-HVD) Chagas Disease outpatient clinic in the state of Amazonas, Brazil. The control group consisted of 31 healthy volunteers with no history of heart disease and no reactive serology for Chagas disease. Baseline ECG was performed in all subjects. The Selvester scoring method was performed according to the standardized guide (< 3 points: no myocardial injury,> 3: points × 3% = % of the predicted LV infarction).

**Results:**

A total of 62 subjects were included, 31 as cases and 31 as controls. The mean follow-up of the case group was 17 months. The control group presented normal ECG. The case group presented 13 alterations before treatment and 11 after. Nineteen individuals presented scores > 3 points, 6 before and 13 after. In 19.36% of subjects, myocardial injury was found before treatment and in 41.94% after treatment.

**Conclusion:**

This is the first study that uses the Selvester score (SS) to predict myocardial injury in subjects with ACD. The results of this study suggest the significant presence of myocardial injury from the beginning of treatment to the period post treatment of ACD, which demonstrates that the SS can be applied for stratification and follow-up of Chagas disease in the Amazon region.

## Background

Chagas disease (CD) is caused by *Trypanosoma cruzi*, a hemoflagellate protozoan described by the Brazilian physician Carlos Chagas in 1909. In addition to the etiological agent, Chagas also described the clinical aspects of the disease and its vector [1]. Currently the disease affects approximately eight million people in the world and about six million of these are in Latin America [2].

The American continent has the greatest contingent, and the vectors are distributed from the southern United States of America to Argentina [3]. In addition to the Americas, CD has been detected in Canada, European countries and some Western Pacific countries. As such, studies indicate the main cause of this spread to population mobility between Latin America and the rest of the world [4].

Considered a neglected disease, CD presents two clinical phases: one, which is acute and characterized by nonspecific clinical manifestations and high parasitemia, and the other, which is chronic [5].

In Brazil, more precisely in the Amazon region of Brazil, in recent decades, there has been an important increase in the number of cases, especially in acute forms, including cases with cardiac impairment, and electrocardiographic and echocardiographic alterations [6].

The chronic phase is subdivided into an asymptomatic form, also called indeterminate, with occurrence of reactive serologies, but without clinical manifestations; a digestive form with manifestations of megaesophagus or megacolon; and a cardiac form, indicated as the main cause of death, due to arrhythmic manifestations or heart failure [7].

Cardiac involvement occurs in all phases of CD, approximately 40% of cases being in the acute phase, where inflammatory disease occurs [8]. With persisting parasitism, it develops to the latent or latent indeterminate phase, where it can present cardiac impairment, even without clinical signs or symptoms. About 10 to 20 years later, 30% of individuals may evolve into the chronic phase, which has greater cardiac impairment, such as dilated cardiomyopathy, and manifests itself as congestive heart failure (CHF), ventricular arrhythmias and, as such, has a significant mortality rate in the first five years [9].

Chronic Chagas heart disease (CCH) presents clinically in the form of three fundamental syndromes: heart failure, arrhythmias and thromboembolis [10]. In CCH, tissue destruction leads to interstitial and focal, and the parasite is limited to the myocardium at this point; therefore, tissue destruction is most likely caused by a combination of autoimmune and inflammatory diseases [11]. This cardiomyopathy is considered to have a much worse prognosis when compared to the others [12]. 

In CCH, ECG alterations are frequent and are considered to be the first indicator of the onset of disease [13]. The ECG of the fragmented surface (QRS) has recently been described as an easy and practical method for predicting the risk of total and arrhythmic death in subjects with coronary artery disease (CAD) [14].

The Selvester score (SS) was developed by Selvester and collaborators in the 1970s, who identified, quantified and located the presence of necrosis and in the myocardium of subjects with ischemic cardiomyopathy [15]. These data were recorded from the ECG and compared with findings in the necropsy of these subjects. In the beginning, the presence of confounding factors in the ECG, such as branch blockages and ventricular hypertrophy, induced error in the analysis of the ventricular depolarization pattern and consequently the alteration of the QRS, since it was known that the presence of myocardial interfered in this process [16].

With the passage of time, new scores were included in the SS to be used in the presence of ECG confounding factors, and these proved effective and were able to identify and quantify myocardial scars in comparison with cardiac resonance in subjects with ischemic and non-ischemic cardiomyopathy, including subjects with chagasic cardiopathy [17, 18].

Given the importance of early detection of these alterations, the SS was developed to record slight alterations in cardiac electrical activity in a simple evaluation of the 12-lead electrocardiogram (ECG) [15], in which it will perform the SS, which estimates the size of the myocardial scar, by alterations in the quantification of the duration, amplitude and morphologies of the Q, R and S waves. Each point of the QRS score corresponds to 3% scarring of the left ventricle, which corroborates for a more accurate early, less invasive and more affordable diagnosis [16].

The direct correlation between SS and myocardial scar is already well established, and the wide availability of 12-lead ECG makes it a very useful screening tool in CD, since it can improve the clinical risk stratification of subjects with this pathology [19].

Another way to identify and quantify the myocardial scar in myocardial of CD is cardiac resonance (CMR) with the presence of late gadolinium enhancement (LGE-CMR), currently considered the in vivo gold standard test [20]. This test provides a good correlation between the degree of myocardial determined by LGE-CMR, and establishes a prognostic factor in coronary artery disease (CAD), supporting the role of LGE-CMR as a marker of the severity of the disease. In addition, it provides evidence of myocardial involvement among CD-positive subjects without clinical symptoms or myocardial contractility dysfunction. The use of LGE-CMR is also supported to define as a subclinical marker of the severity of Chagas disease, however, it is a high-cost examination, most often inaccessible to most of those who need it [19, 20].

Thus, the SS has become a viable, interesting and useful alternative to evaluate and quantify myocardial injury in subjects with the various evolutionary forms of CD, since many, such as indigenous subjects from the Amazon have difficulties in accessing CMR, either because of costs or geographical isolation. The SS is a cheap method, has good availability and rapid analysis and is precise when well employed by well-trained examiners. The advantage that this clinical method can offer is that it can meet the demand of subjects affected with CD, and, with this, track and monitor at low cost all those who have risks of symptomatic progression to increased SS cardiomyopathy, and then direct them to the most appropriate and specific treatment for their type of cardiac complication [18].

The increase in the number of acute cases in the Amazon and the peculiar epidemiological profile of a region with difficulty in accessing more complex examinations, such as CMR, contributed to the objective of this study, which is to evaluate in subjects with CAD before and after treatment through the use of the Selvester score.

## Methods

### Study design

This is a longitudinal study of subjects evaluated during the acute phase of Chagas disease, before treatment and followed-up in a mean period of 17 months (12–23) after treatment.

### Study area

The study was carried out in Manaus**,** Amazonas state capital (Brazil), at the Tropical Medicine Foundation Dr. Heitor Vieira Dourado (FMT-HVD), which is a tertiary care center specialized in infectious diseases.

### Study population

Indigenous subjects from the Amazon region, in clinical follow-up at the FMT-HVD Chagas disease outpatient clinic, and who were evaluated in the pre-and post-treatment phases. The inclusion criteria were as follows: subjects being from the Brazilian Amazon, with a positive direct parasitological examination (blood smear). Those with previous acute myocardial infarction (AMI), coronary artery disease (CAD), more than two risk factors for CAD, valvular disease and previous trips to other transmission areas outside the Brazilian Amazon were excluded from the study.

The control group included healthy volunteers who had no history of heart disease and non-reactive serology for Chagas disease, who were matched by sex and age with the case group.

### Electrocardiographic analysis (GLOBAL)

All subjects underwent a standard 12-lead ECG (10 mm/mV and 25 mm/s). The analysis of the ECGs was performed blindly by two experienced cardiologists, without knowledge of the purpose of the study, who determined whether or not there were signs of defects in electrocardiographic conduction. No clinical data other than ECG tracing were provided, nor were guidelines given on the specific criteria to be sought. Instead, they based their assessment on clinical experience, looking for atrial overload, branch blockage, overload, and ventricular hypertrophy. The differences found between the evaluations of the two cardiologists were judged at the meetings of the research group so that each individual score could be obtained. In addition to evaluation by cardiologists who were not related to or involved in the research, ECGs were also evaluated by group members based on the criteria established in the Brazilian electrocardiogram guidelines [21]. Thus, the ECGs were classified by type of ventricular conduction/hypertrophy as follows: left branch blockage (LBB), left anterior fascicular blockage (LAFB), left ventricular hypertrophy (LVH), right branch blockage (RBB), RBB + LAFB and absence of confounding factors. The overload of the right atrium was also classified, since it has an important implication at the time when it should be scored in the V1 and V2 leads [18].

### Selvester QRS score

The evaluation of the Selvester score (SS) followed the guidelines established by Loring et al. (2011) [18]. The score was then applied to the appropriate type of conduction/hypertrophy, which involves measurements of the amplitude, duration and notches of the Q, R and S waves. Each QRS point represents a scar size involving 3% of the total mass of the LV. The score allows an estimate of the size of the affected area, since it is based on a quantitative assessment of changes in QRS waves related to myocardial scars (possibly fibrous regions in cardiac tissue). The scoring system is based on the criteria for 10 of the 12 leads on a standard 12-lead ECG (aVL, aVF, I, II, V1 - V6). Points were awarded for the duration of the Q wave; amplitude and duration of the R wave; and R/S or R/Q ratios. Two observers completed the score blindly assigning points using the QRS waves of each subject, before and after treatment and for the control group. Given the knowledge of ECG reports, both observers adjusted the amplitude and duration of the QRS waves by sex and age in order to correspond to the voltages, which in younger men are higher, and in older women are lower as proposed by Loring et al., 2011 [18]. For amplitude of QRS adjust adding 1% per year when age 20 to 54 years old; reducing 1% per year when > 55 years old; reducing 10% for females) and for duration of the QRS adjust reducing 10% for females. Afterwards, the differences in points and percentages obtained by the two observers were compared during a meeting, in which no disagreements were detected. The percentage of left ventricular scars estimated by ECG was calculated by multiplying the overall QRS score by three, since each point was designed to correspond to approximately 3% of left ventricular impairment.

### Ethical considerations

All participants signed an informed consent form and the study was approved by the Research Ethics Committee at the FMT-HVD, under approval number 66077017.8.0000.0005/2.043.174, in accordance with resolution 466/12 of the Brazilian National Health Council and the ethical guidelines of the 1975 Helsinki Declaration.

### Presentation and analysis of data

The data are described and presented in a Table as mean ± SD of the variables for age, score and prediction of myocardial injury. For the analysis of these variables, the ANOVA test (unidirectional variance analysis) was used and the subsequent Tukey test for comparison with the control group was performed using Stata/MP v. 13.0.

## Results

A total of 62 participants were included, 31 with ACD, with a mean follow-up period of 17 months and a mean age of 40 ± 17 years, and 48 (52%) subjects were males. The pairing between age and sex was performed in order to minimize confounding factors in the evaluation of the score (Table 1).

**Table 1:** Subjects baseline characteristics

Variable	Total	Control group	ACD Cases group			***p***-value
	(***n*** = 62)	(*n* = 31)	(***n*** = 31)	Before Treatment	After Treatment	
**Male**	32 (52%)	16 (52%)	16 (52%)			1.000†
**Female**	30 (48%)	15 (48%)	15 (48%)			
**Age (y)**	40 ± 17	43 ± 16	38 ± 19			0.454**
**QRS scoring**	1.2 ± 1.9	0,06 ± 0.36		1.1 ± 1.5‡‡	2.6 ± 2.2‡‡	< 0.001‡
**Predicted LV infarct (%)**	3.7 ± 5.5	0.2 ± 1.1		3.3 ± 4.5‡‡	7.7 ± 6.6‡‡	< 0.001‡
**LVEF (%)**	73 ± 6.3	75 ± 4.6	73 ± 7.3			0.092‡
**NYHA I**	61 (98.4%)	31 (100%)		30 (97%)	31 (100%)	
**NYHA III**	1 (1.6%)			1 (3%)		

*LV* left ventricular, *LVEF* left ventricular ejection fraction, *NYHA-FC* New York Heart Association Functional Classification. Data are expressed as mean ± SD. In parenthesis is the percentage of the total group. **One-way analysis of variance by ranks-Kruskal-Wallis test. † Pearson’s chi-squared. ‡ One-way analysis of variance (ANOVA). ‡‡ p < 0.05 Tukey test for comparisons with control group

### Electrocardiogram analysis (GLOBAL)

In 13 (42%) of the subjects in the pre-treatment ACD group, the ECG was altered. The abnormalities found were ventricular repolarization alterations (VRA) in 7 (54%), RBBB + LAFB in 2 (15%); only RBBB, right branch conduction disorder (RBBCD), low voltage and ventricular extrasystole in 1 (8%) of each. In subjects in the group with ACD, after treatment, ECG was altered in 10 subjects (32%). Among the abnormalities were VRA in 4 (40%), RBBB + LAFB and RBBCD in 2 (20%) of each; and ventricular and supraventricular extrasystole in 1 (10%) of each.

### Selvester QRS score

Regarding the evaluation of the Selvester score, it was observed that, in the control group, all individuals had a score of 2 and were considered as not having myocardial injury. Subjects in the ACD group underwent the application of the Selvester score in the following two stages: before the start of antiparasitic treatment with benznidazole (Rochagan®) and after treatment (average of 17 months). For the 31 subjects evaluated before treatment, 6 individuals had a score above 3, showing the presence of myocardial injury in 19.36% of this group (Table 2). However, in the post-treatment period, 13 individuals presented scores above 3, which represents the presence of myocardial injury in 41.94% of this group. There was an expressive score for myocardial injury, both in the initial acute phase and in the post-treatment group. The mean score of the pre-treatment acute CD group, when compared to the control group, was statistically significant (1.1 vs. 0.06, *p* = 0.018), as well as of the post-treatment ACD group, compared to the control group (2.6 vs. 0.06, *p* < 0.001). When the ACD group was compared according to the time of initiation of benznidazole administration, it was also statistically significant (1.1 vs. 2.6, *p* = 0.011) (Table 3).

**Table 2:** Individual predicted LV infarct (%) before and after treatment

ID	Predicted LV infarct (%)		Follow-up period (months)
	Pre-treatment	Post-treatment	
Subject 01	3	9	15
Subject 02	6	6	15
Subject 03	18	18	16
Subject 04	0	0	18
Subject 05	6	9	20
Subject 06	0	9	21
Subject 07	3	6	21
Subject 08	9	0	20
Subject 09	0	6	13
Subject 10	0	6	18
Subject 11	0	0	17
Subject 12	0	0	16
Subject 13	0	18	17
Subject 14	0	9	15
Subject 15	3	0	14
Subject 16	6	6	14
Subject 17	3	18	12
Subject 18	0	6	15
Subject 19	0	6	19
Subject 20	9	18	18
Subject 21	0	0	17
Subject 22	12	6	18
Subject 23	0	12	14
Subject 24	0	9	20
Subject 25	0	0	23
Subject 26	0	0	20
Subject 27	9	24	18
Subject 28	0	15	12
Subject 29	9	6	16
Subject 30	3	6	18
Subject 31	3	12	13

*LV* left ventricular

**Table 3:** QRS scoring and predicted LV infarct (%) between groups

	Control vs. Pre-treatment	*p*-value	Control vs. Post-treatment	*p*-value	Pre-treatment vs. Post-treatment	*p*-value
**QRS scoring**^a^	0,06 ± 0.36 vs. 1.1 ± 1.5	0.018	0,06 ± 0.36 vs. 2.6 ± 2.2	< 0.001	1.1 ± 1.5 vs. 2.6 ± 2.2	0.011
**Predicted LV infarct (%)**^a^	0.2 ± 1.1 vs. 3.3 ± 4.5	0.021	0.2 ± 1.1 vs. 7.7 ± 6.6	< 0.001	3.3 ± 4.5 vs. 7.7 ± 6.6	0.008

*LV* left ventricular. ^a^Student’s t-test

## Discussion

This is the first study on the evaluation of the Selvester score in subjects with acute Chagas disease. Historically, the Brazilian Amazon was considered a non-endemic region for Chagas disease and classified as an area of low morbidity and mortality. In recent years, the number of cases of ACD has increased [2], although still little is known about the clinical outcome of the disease in our region, some studies have been reported cardiological, serological and responses to treatment [6, 22]. Studies conducted in the state of Pará, evaluating acute cases of CD in a follow-up of up to 9 years, found no signs of chronic disease in the cases followed and the Selvester score, which is indicative of myocardial injury for cases of CD, was not used. In Amazonas, studies accompanying patients with CD are more recent [6, 23] and circulating *T. cruzi* has been identified in both acute [24] and chronic disease [25], information that is not yet known in others state of Amazon region. This is due to the fact that most subjects come from remote areas, far from specialized diagnostic and treatment services, and for even those who manage to reach these centers, follow-up is impaired and is often not carried out, since subjects after returning to their homes, rarely return to the service for follow-up which constitutes a limiting factor in the handling of cases.

Several studies have shown that the presence of myocardial fibrosis is associated with a worse cardiac outcome, regardless of etiology [26, 27]. Volpe et al. (2018) demonstrated similar occurrences in subjects with CCH, with worse results observed in the group with evidence of fibrosis through CMR [19]. In a series of subjects, Rochitte et al. (2005) demonstrated that myocardial fibrosis was present in 84.6% of subjects with chronic chagasic heart disease and in 100% of subjects with chronic chagasic heart disease and sustained tachycardia [28].

In our study, although acute subjects present with more myocardial injury after initial treatment, we also observed this injury in pre-treatment phase, ACD causes acute inflammation of the myocardium, and this process can trigger the replacement of heart tissue with non-functional structures, that is not always considered as fibrosis. These injuries may include edema, necrosis, inflammatory interstitial infiltrate and myocytolysis [29].

It is important to emphasize that Souza et al. (2017) observed only myocardial edema, without myocardial fibrosis, through cardiac resonance, but in only one subject with acute Chagas disease [30].

The presence of more significant myocardial injury in the post-treatment phase, compared to the pre-treatment phase, suggests that, despite the good therapeutic response from the clinical point of view, myocardial tissue is replaced by fibrotic tissue. This reinforces the need for long-term follow-up of these subjects and the need for more detailed studies, such as those using CMR.

The Selvester score, however, may be a first step in the stratification of risk of subjects with ACD, at least in the medium-term follow-up, thus allowing an earlier intervention and minimizing the deleterious effects of the disease.

These data can be compared with the TIMIC study [31], in which subjects with acute myocarditis were treated with immunosuppressive drugs and showed significant improvement in clinical parameters and ventricular function. However, in myocardial biopsy, a replacement of inflammatory alterations is found in the myocardium through the presence of fibrotic tissue.

This study shows that the application of the Selvester score can be an alternative tool in the early evaluation and prognosis of these subjects in relation to myocardial injury. However, public health policies that implement screening and monitoring of subjects diagnosed and treated for MF in this phase of CD are needed.

As limitations of this study, we highlight the failure to perform CMR, however, the use of the Selvester score, although not a substitute for CMR, can serve as a screening tool for more severe cases. CMR studies will be developed in our group, in subjects with CD that are native to the Amazon, and will be used to follow up the investigation of myocardial fibrosis in this group of subjects.

## Conclusions

In this study, the evaluation of myocardial injury using the Selvester score in acute Chagas disease showed estimated frequencies of 19.36 and 41.94% before and after benznidazole treatment, respectively. This is an unprecedented finding that draws attention to the probable use of this tool in ACD in the Brazilian Amazon, which can impact the evaluation, follow-up and outcome of cases, including sudden death, depending on the degree of cardiac injury found.

## Data Availability

The datasets used and/or analysed during the current study available from the corresponding author on reasonable request.
